# Violence Affects Physical and Mental Health Differently: The General Population Based Tromsø Study

**DOI:** 10.1371/journal.pone.0136588

**Published:** 2015-08-28

**Authors:** Oddgeir Friborg, Nina Emaus, Jan H. Rosenvinge, Unni Bilden, Jan Abel Olsen, Gunn Pettersen

**Affiliations:** 1 Department of Psychology, Faculty of Health Sciences, UiT The Arctic University of Norway, Tromsø, Norway; 2 Department of Health and Care Sciences, Faculty of Health Sciences, UiT The Arctic University of Norway, Tromsø, Norway; 3 Department of Community Medicine, Faculty of Health Sciences, UiT The Arctic University of Norway, Tromsø, Norway; Univ of Toledo, UNITED STATES

## Abstract

This general population-based study examined associations between violence and mental health, musculoskeletal pain, and early disability pension. The prevalence and consequences of good vs. poor adjustment (resilience vs. vulnerability) following encounters with violence were also examined. Data were based on the sixth wave of the “Tromsø Study” (*N* = 12,981; 65.7% response rate, 53.4% women, *M*-age = 57.5 years, *SD*-age = 12.7 years). Self-reported data on psychological (threats) and physical violence (beaten/kicked), mental health (anxiety/depression), musculoskeletal pain (MSP), and granting of disability pension (DP) were collected. Men suffered more violent events during childhood than women did, and vice versa during adulthood. Psychological violence implied poorer mental health and slightly more MSP than physical violence. The risk of MSP was highest for violence occurring during childhood in women and during the last year for men. A dose-response relationship between an increasing number of violent encounters and poorer health was observed. About 58% of individuals reported no negative impact of violence (hence, *resilience* group), whereas 42% considered themselves as more vulnerable following encounters with violence. Regression analyses indicated comparable mental health but slightly more MSP in the resilience group compared to the unexposed group, whereas the vulnerable group had significantly worse health overall and a higher risk of early granting of DP. Resilience is not an all-or-nothing matter, as physical ailments may characterize individuals adapting well following encounters with violence.

## Introduction

The human and societal tolls of violence are substantial. In 2002, the World Health Organization published the first report on violence and health [1], also highlighting the considerable benefits for the public health sector in taking an active role in prevention. Universal prevention is generally the best strategy, that is, finding sociopolitical interventions that target the roots or causes of violence in order to prevent its occurrence. Nonetheless, the role of public health services is of utmost importance in reducing the immediate or long-term health consequences among people exposed to violence.

Violence comes in many forms, such as self-directed (e.g., self-harm or suicidal attempts), interpersonal (e.g., family/intimate partner violence), or collective (e.g., politically or economically motivated). More distressing, however, is violence that is inflicted in close relationships and involves more force or is endured for a longer time [2]. Violent events that are interpersonal and intentional (e.g., torture, rape, abuse, or bullying) are more difficult for people to cope with compared to non-intentional events, such as serious accidents [3]. Violent events may involve either threats or actual violence, or a combination of both. Although physical injuries may ensue and add to the psychological burden after physical violence, threats of violence may represent a larger psychological burden than actual violence, as posed threats usually are not limited in time. Although physical violence may also involve threats, they usually are constricted in time and by the context. Studies on post-traumatic stress disorders support this distinction, as threat appraisals seem to mediate the degree of adjustment problems following trauma exposure [4, 5]. However, to the best of our knowledge, no general population-based studies have examined this distinction. In the present population-based study, two questions asking about exposure to violence (threats vs. actual violence) were included, thus allowing an analysis of their respective contribution to health problems.

Exposure to violence carries numerous potential consequences spanning three overarching domains: increased mortality, morbidity, and disability [1, 6–8]. Increased psychological morbidity, most notably as depression, suicidality, substance abuse or post-traumatic stress disorders is common subsequent to experiencing interpersonal violence [9–11]. Chronic functional problems following such events, such as higher risk of disability pension [8] are also elevated. A more general yet overlapping term in the literature that encompass a wider range of adverse life events, including violence, trauma, losses, bereavement, or illnesses, are *negative life events* (NLEs). NLEs seem to be sturdy predictors for later health problems, although the relationship generally is weaker than for violence [9]. NLEs have been independently associated with recurrent pain problems [12], acute infections [13], myocardial infarction [14], cardiovascular diseases [15], colorectal cancer [16], or skin diseases [17]. Comparably, NLEs increase the risk for mental health problems [13] as well as major depression [18] and schizophrenia [19]. NLEs, in particular violence, may also disrupt concentration and memory and indirectly impair academic performance [20, 21] contributing to early disability pension [22].

Experiencing violence may affect women and men differently, as reported in a large Swedish population study (*N*~6,100), which showed that women had doubled the odds of poorer psychological health than men [23]. A meta-analysis on domestic violence confirmed that subsequent internalizing symptoms (e.g., depression) are more common among girls than boys [24]. Comparable gender differences have been reported in other studies [25], which also reported that men may suffer from somatic health problems more often than women do.

### 1.1 Resilience to NLEs

A popular myth about exposure to violence or trauma is that subsequent health problems will follow. The extensive search for risk factors is a testimony of this orientation also among researchers. However, the majority of victims seem to cope or function relatively well in the aftermath of trauma [26, 27]. This outcome has been termed *resilience* [28–30] and has been defined as a relatively good outcome (no pathology) or a relatively normal functioning despite exposure to significant stressors, violence, or trauma. In the present population-based study, a question about the degree of negative impact subsequent to exposure to violent events was included, hence making an analysis of resilience possible, as the large sample size compensated for the reduced accuracy and statistical power of using single questions. As resilience may be defined as “no pathology” or relatively “normal functioning”, we used responses to the impact question to create subgroups describing unexposed subjects (no violent exposure), resilient (exposed but good adjustment), and vulnerable (exposed but poor adjustment) subjects.

The adaptation process may, however come at a price, as the higher *allostatic load* caused by the “wear and tear” of NLEs [31] may cause neuroendocrine and immunological changes that over time may lead to somatic health complaints or diseases [32]. Subjects characterized as resilient are expected to experience less “wear and tear” as they generally cope better with NLEs than vulnerable subjects. However, individuals with a history of childhood maltreatment, but who are considered resilient (good mental health), seem to show more blunted adrenocorticotropic hormone and cortisol responses [33] and larger immunological responses [34] to later minor stressors than unexposed healthy individuals do. Subjects that cope poorly show stronger corticosteroid and inappropriate inflammatory responses, thus experiencing even more “wear and tear” [35]. However, the tendency for a stronger neuroendocrine and immunological response among those considered resilient may therefore enhance the vulnerability for somatic health problems over time, which are quantifiable as symptoms of musculoskeletal pain.

### 1.2 Aims of the study

The large sample size in the “Tromsø study” (N = 12,981) makes it possible to examine whether subjects classified as resilient show different profiles with regard to job functioning and, mental and musculoskeletal health complaints compared to unexposed subjects. Moreover, by using general population-based data, gender differences similar to those reported by Schlack and Petermann (25) could be examined.

We pose several hypotheses: 1) psychological violence is associated with worse ratings of mental and physical health as well as job functioning, 2) subjects considered as resilient are expected to show equally good mental health and job functioning as unexposed subjects but more health complaints in terms of musculoskeletal pain due to the increased allostatic load caused by psychological violence in particular, and 3) subjects more vulnerable to exposure to violence are expected to demonstrate the poorest health and job functioning.

In addition, a range of demographic data as well as alcohol use are associated with exposure to violence [36], thus these covariates were included in the statistical analyses to adjust for their effects. Violence does not happen entirely at random, rather, those who are younger, unmarried, with lower education and income, in unskilled jobs or consuming more alcohol are more frequently perpetrated [1, 36]. The prevalence of health problems, similarly to those measured in the present study, is also higher in these subgroups [37, 38], which strengthens the argument for adjusting for these variables. Moreover, as obesity [39] and lack of physical activity [40] are well-known risk factors for musculoskeletal health problems, information about body composition (body mass index, BMI) and self-reported physical activity were also included.

## Materials and Methods

The “Tromsø study” is a longitudinal general population-based multi-purpose study focusing on lifestyle-related diseases [41]. The study consisted of six waves starting in 1974 and was repeated last time in 2007/08 [42]. All surveys included questionnaires and various clinical measurements. The present study is based on data collected in the 2007/08 wave, which for the first time included questions on violence exposure. Altogether 19,762 subjects were invited and 12,984 (65.7%) participated, 6,054 men (62.9%) and 6,930 women (68.4%). Mean age was 57.5 years (*SD* = 12.7).

The Regional Committee of Medical and Health Research Ethics and the Norwegian Data Inspectorate approved the study. Each participant gave written informed consent prior to inclusion.

### 2.1 Questionnaire

Along with the invitation letter, subjects received the first questionnaire (Q1), which they completed at home and brought to the study site. Here, a research technician checked it for inconsistencies and incomplete data. The participants were then given a second questionnaire (Q2), which they could fill in at the site or at home. The Q2 asked for two kinds of experiences with violence: i) *Have you experienced being tormented or threatened with violence over a long period of time*? and ii) *Have you been beaten*, *kicked or been victim of other types of violence over a long period of time*? The subjects could tick any of the following answers: 0-No; 1-Yes, experienced as child; 2-Yes, experienced as adult; 3-Yes, experienced last year. Following these questions, the subjects reported the negative impact of these events on their life (1-not affected, 2-affected to some extent, or 3-affected to a large extent). This question was used to classify the subjects’ response as indicating vulnerability or resilience following exposure to these events.

In addition, the Q2 included the following question about musculoskeletal complaints: *Have you suffered from pain and/or stiffness in muscles and joints lasting for at least 3 months during the last year*? The subjects were given three response alternatives: 0-no complaints, 1-little complaints, and 2-severe complaints across six sites (pain in neck, arms, hip, upper back, lumbar, or other sites). Symptoms of mental health problems were assessed in the Q1 using a short version of the Hopkin’s Symptom Check List (HSCL, 90 items), that is, the HSCL-10, which is a widely used, self-administered instrument measuring psychological distress in population surveys. Reponses are recorded on a 4-point scale (1-not at all, 4-very much), and the average score is calculated. Higher scores indicate more symptoms, and scores above 1.75 indicate significant depression requiring treatment [43].

The following covariates from Q1 and Q2 were included: age, BMI (weight kg/height m^2^), marital status (single, married/cohabitating, widow(er), or divorced), educational status (primary, high school, or university), occupation (unemployed, retired, part-time, or full-time), household income (equivalent in USD, classified as low <49,425, medium 49,425–90,311, or high > 90,311; according to official categories of Statistics Norway in 2007), physical activity (low, moderate, or high), smoking status (never, former, or present smoker), and frequency (never/rarely, weekly or more) and amount (1–2, 3–6, or 7+ weekly units) of alcohol use.

### 2.2 Statistics

Statistical analyses were conducted using SPSS version 21. Univariate gender differences were examined with independent t-tests for continuous variables and chi-square tests for dichotomous variables. Hierarchical linear regression analyses were used to examine the association between violence and mental distress scores (HSCL-10). Childhood violence was entered first, adulthood violence second, and last year violence third, thus separating their explanatory contributions. The covariates were entered in the last step to provide crude and adjusted coefficients for the violence variables. The following variables were categorical and hence dummy coded with zero as the reference category: *marital status* (0-married, 1-single, 2-widow(er) and 3-divorced), *level of education* (0-college/university, 1-upper secondary school and 3-primary school), *occupation* (0-full time, 1-part time, 2-retired and 3-unemployed), *household income* (0-high, 1-medium and 2-high), *degree of exercise* (0-high, 1-medium and 2-low), and *units of alcohol* (0-low, 1-medium and 2-high). All other variables were either continuously or binary coded (0/1). The HSCL-10 variable was log-transformed as it was positively skewed (Z) and lepto-kurtotic, which is normal for low prevalent phenomena. A similar regression approach was used for the resilience classification variable, which was entered first and then adjusted by number of violent events and covariates in separate steps. The six musculoskeletal pain variables were summed and treated as a count variable (range 0–6, *M* = 1.44, *SD* = 1.50). The mean and SD values were almost similar, and thus a Poisson regression analysis is recommended [44]. The log link function and robust standard errors were used to produce error bands for the Wald statistics. All analyses were stratified by gender. The alpha-level was set to .05. A logistic regression analysis was set up similarly for the granting of disability pension (0-no/1-yes).

## Results

Baseline descriptive characteristics of the study cohort are presented in Table 1, which also gives test statistics for gender differences.

**Table 1:** Characteristics of the Study Cohort.

	Women *n* = 6,928	Men *n* = 6,053	*t* or χ^2^
Continuous variables			
Age, years (SD)	57.8 (13.0)	57.6 (12.3)	ns
Height, cm (SD)	163.3 (6.5)	176.9 (6.9)	111.94***
Weight, kg (SD)	70.9 (13.0)	85.4 (13.3)	61.06***
BMI, kg/m² (SD)	26.6 (4.7)	27.3 (3.7)	8.88***
Dichotomous variables	% (n)	% (n)	
Marital status			
single	16.6 (1147)	19.1 (1154)	13.94***
married	54.1 (3749)	64.9 (3927)	154.63***
widow(er)	13.3 (922)	3.4 (205)	401.11***
divorced	16.0 (1110)	12.7 (767)	29.32***
Educational status			
primary	31.5 (2179)	24.7 (1494)	73.0***
high school	31.3 (2170)	35.0 (2119)	19.83***
university	35.7 (2474)	39.0 (2362)	15.16***
Occupation			
full time	43.3 (3001)	57.6 (3484)	262.08***
part-time	12.6 (871)	5.2 (317)	209.05***
retired	40.8 (2830)	36.6 (2215)	24.62***
unemployed	0.7 (47)	1.1 (66)	6.35*
Economy			
< 49425 USD	26.2 (1818)	18.8 (1137)	102.18***
49425–90311	28.1 (1948)	30.8 (1865)	11.30***
> 90311	34.8 (2413)	46.0 (2786)	168.69***
Physical activity			
low	35.7 (2471)	47.1 (2848)	173.11***
moderate	39.3 (2723)	35.6 (2152)	19.39***
high	21.6 (1496)	15.2 (923)	85.77***
Smoking status			
present	21.0 (1454)	19.1 (1156)	7.18**
former	37.5 (2601)	46.4 (2806)	103.26***
never	39.7 (2748)	33.4 (2019)	55.35***
Alcohol use			
never/rarely	79.0 (5471)	74.7 (4521)	33.38***
weekly	19.1 (1326)	24.3 (1472)	51.24***
Alcohol units			
1–2	61.7 (4274)	47.5 (2874)	263.67***
3–6	21.2 (1469)	39.4 (2382)	509.95***
7 or more (n)	0.6 (45)	4.0 (244)	169.70***

*Notes*. *t* = Student t-tests for continuous variables, and χ^2^ = chi-square tests for dichotomous variables.

*p < .05,

**p < .01

***p < .001.

The degree of exposure to violence is presented in Table 2 for women and men separately. During *childhood*, psychological violence (threatened/feeling tormented) was more common than actual violence. Men reported threats and actual violence significantly more often than women. During *adulthood*, these gender differences went in the opposite direction, with women reporting significantly more experiences with threats and actual violence than men. The prevalence of violence during the last year was low and comparable between women and men.

**Table 2:** Exposure to Violence During Childhood, Adulthood and Last Year.

	Women *n* = 6928	Men *n* = 6053	χ^2^
Childhood			
Tormented/threatened	7.9% (549)	10.0% (608)	17.89***
Beaten/kicked	4.6% (322)	7.9% (478)	58.97***
Any one	9.4% (651)	12.9% (780)	40.11***
Both events	3.2% (220)	5.1% (306)	29.37***
Adulthood			
Tormented/threatened	7.5% (522)	3.5% (211)	99.40***
Beaten/kicked	5.2% (360)	2.4% (148)	65.03***
Any one	9.0% (621)	4.9% (648)	82.40***
Both events	3.8% (261)	1.1% (64)	97.20***
Last year events			
Tormented/threatened	1.4% (100)	1.2% (70)	2.06
Beaten/kicked	0.5% (34)	0.3% (21)	1.58
Any one	1.6% (112)	1.3% (78)	2.41
Both events	0.3% (22)	0.2% (13)	1.27

*Notes*. The number of cases is given in parentheses.

***p < .001.

### 3.1 Relationships between violence, mental health, and musculoskeletal pain

The crude and adjusted coefficients were quite comparable (Table 3). The magnitude of the coefficients (or effect sizes) was small. Psychological violence (threatened/tormented) was associated with worse mental health (HSCL-10) than actual violence, which was a consistent finding independent of gender and life epoch (childhood/adulthood). This picture was to some extent comparable for symptoms of musculoskeletal pain (MSP), except psychological and actual violence were similarly associated with MSP for childhood events in men and adulthood events in women. Regarding recent violent events, men exposed to threats had more MSP than men exposed to actual violence.

**Table 3:** Regression Analyses with Mental Distress (HSCL-10) and Number of Severe Musculoskeletal Pain Sites (MSP) as Outcome.

	HSCL-10 (continuous range 1–4)				MSP (count range 0–6)			
	Women *n* = 6928		Men *n* = 6053		Women *n* = 6928		Men *n* = 6053	
	Crude β	Adj β (CI 95%)	Crude β	Adj β (*CI* 95%)	Crude OR	Adj OR (*CI* 95%)	Crude OR	Adj OR (*CI* 95%)
Childhood events		*R* ^*2*^ = .039		*R* ^*2*^ = .035				
Tormented/threatened	.178***	**.153** *** (.124–.182)	.146***	**.123** *** (.094–.153)	1.26***	**1.31** *** (1.20–1.42)	1.09	**1.13** * (1.02–1.26)
Beaten/kicked	.038*	**.010** (-.018–.038)	.063***	**.052** *** (.023–.081)	1.10	**1.05** (.95–1.16)	1.21***	**1.19** **(1.07–1.33)
Unexposed						1 (ref)		1 (ref)
Adulthood events		*R* ^*2*^ = .068		*R* ^*2*^ = .056				
Tormented/threatened	.167***	**.129** *** (.098–.159)	.152***	**.113** *** (.086–.140)	1.16**	**1.17** *** (1.06–1.28)	1.32***	**1.22** ** (1.06–1.40)
Beaten/kicked	.055***	**.028** (-.002–.058)	.040**	**.005** (-.019–.029)	1.16**	**1.12** * (1.01–1.25)	1.08	**1.07** (.89–1.30)
Unexposed						1 (ref)		1 (ref)
Last year events		*R* ^*2*^ = .075		*R* ^*2*^ = .067				
Tormented/threatened	.124***	**.088** *** (.061–.115)	.134***	**.113** *** (.086–.140)	1.11	**1.09** (.90–1.31)	1.54***	**1.53** ** (1.24–1.88)
Beaten/kicked	-.023	**-.031** * -(.057–.004)	-.019	**-.030** * -(.057–.003)	1.26	**1.25** (.91–1.71)	.93	**.86** (.53–1.39)
Unexposed		*R* ^*2*^ = .118		*R* ^*2*^ = .119		1 (ref)		1 (ref)
Covariates ^1^								

*Notes*. The HSCL-10 was log transformed.

* *p* < .05,

** *p* < .01

*** *p* < .001.

β = Standardized linear beta-weight, OR = Odds-ratio (Poisson regression) with unexposed as reference. The crude and adj β columns represent the coefficients at the first and final blocks, respectively.

^1^ The fully adjusted model (columns adj β/adj OR) included the following covariates: age, BMI, BMI^2^, marital status, educational status, occupation, socio-economy, physical activity, alcohol use (frequency and amount). *R* ^*2*^ = adjusted *R*-square (explained variance).

*Adjusted analysis*: A range of covariates were included (see note of Table 3). With regard to mental health (HSCL-10), BMI, occupation status and age contributed most. BMI showed a curvilinear relationship (♀β = -.250** and β^2^ = .255**; ♂β = -.254* and β^2^ = .268*) indicating poorer mental health for lower and higher BMIs. Being retired (♀β = .152***, ♂β = .1117***), older (♀β = -.109***, ♂β = -.145***), low socio-economic status (♀β = .097***, ♂β = .133***) and low physical activity (♀β = .095***, ♂β = .053**) were associated with poorer mental health.

With regard to musculoskeletal pain (MSP), the strongest contributors (in terms of Wald and OR) were level of education (primary school ♀OR = 1.26*** and ♂OR = 1.30***, and high school ♀OR = 1.18*** and ♂OR = 1.20*** compared with university education), occupation status (part-time ♀OR = 1.17*** and ♂OR = 1.14***, and retired ♀OR = 1.17* and ♂OR = 1.08 compared with full-time), age (♀OR = 1.002 and ♂OR = 1.008***), BMI with a curvilinear relationship (♀OR = 1.08***/OR^2^ = .99** and ♂ OR = 1.01/OR^2^ = 1.00) and low level of physical activity (♀OR = 1.08* and OR = 1.08).

### 3.2 Adjustment to violence and health status

By using the variable assessing the negative impact of violence (see Method section 2.1 Questionnaires), four clusters indicating the adjustment status subsequent to experience with violence, or the degree of *resilience*, could be created. The cluster not reporting any exposure to violence represented the majority (0-unexposed group, *n* = 9,103, 70.1%). Among the subjects reporting violence, three clusters were identified: 1-resilient (experienced violence but no negative impact, *n* = 2,255, 58.1%), 2-vulnerable (experienced violence and affected by it to some extent, *n* = 1,451, 37.4%), and 3-highly vulnerable (experienced violence and largely affected by it, *n* = 172, 4.4%). The gender differences (women vs. men) for the unexposed (52% vs 48%) and the resilient (49% vs. 51%) groups were minor, but were significant for the vulnerable (66% vs. 34%, *p* < .001) and highly vulnerable (75% vs. 25%, *p* < .001) groups.

The crude analysis indicated considerably worse mental health status in the vulnerable clusters compared to the unexposed or resilient clusters (Table 4). The resilient cluster had slightly worse mental health than the unexposed cluster. Adjusting for the number of violent events maintained the findings; however, the minor difference in mental health between the resilient and unexposed cluster disappeared. Including all the covariates in the model did not change this finding. A final noticeable finding was that the number of experienced psychological threats (from childhood and adulthood to recently, range 0–3 events) significantly predicted poorer mental health, while the number of *physical* violent events had no contribution.

The regression model for musculoskeletal pain (MSP) painted a rather similar picture, except that the resilient group had significantly more pain symptoms than the unexposed group but less than the vulnerable groups that had the highest MSP scores. Again, a higher number of experienced threats predicted more MSP than a higher number of actual occurrences of violence.

**Table 4:** Regression Analysis Examining the Relationship Between Adaptation Status, Mental Distress and Severe Musculoskeletal Pain.

	HSCL-10 (continuous range 1–4)						MSP sites (count range 0–6)					
	Women *n* = 6928			Men *n* = 6053			Women *n* = 6928			Men *n* = 6053		
	Β	β	Adj β (*CI* 95%)	β	Β	Adj β (*CI* 95%)	OR	OR	Adj OR (*CI* 95%)	OR	OR	Adj OR (*CI* 95%)
Adaptation status			*R* ^*2*^ = .103			*R* ^*2*^ = .096						
Resilient	.049***	-.010	**.003** (-.024–.029)	.069***	-.014	**-.007** (-.038–.023)	1.14***	1.09**	**1.14** *** (1.07–1.22)	1.16***	1.11*	**1.15** *** (1.06–1.25)
Vulnerable	.222***	.143***	**.145** *** (.116–.173)	.263***	.196***	**.186** *** (.158–.214)	1.32***	1.24***	**1.30** *** (1.21–1.39)	1.38***	1.30***	**1.31** *** (1.19–1.45)
Highly vulnerable	.252***	.211***	**.203** *** (.178–.228)	.176***	.148***	**.141** *** (.115–.166)	1.47***	1.36***	**1.31** *** (1.14–1.51)	1.72***	1.58***	**1.49** ** (1.13–1.96)
Unexposed (ref)									1 (ref)			1 (ref)
No. events (range)			*R* ^*2*^ = .125			*R* ^*2*^ = .113						
Threats (0–3)		.177***	.168*** (.135–.201)		.148***	.137*** (.104–.169)		1.06*	1.11*** (1.05–1.17)		1.05	1.09* (1.00–1.18)
Physical (0–3)		.006	-.009 (-.039–.021)		.035*	.024*** (-.006–.054)		1.06	1.05 (.98–1.12)		1.07	1.07 (.98–1.17)
Covariates ^1^			*R* ^*2*^ = .163			*R* ^*2*^ = .158						

*Notes*. The HSCL-10 (Hopkins Symptom Check List) variable was log transformed.

* *p* < .05,

** *p* < .01

*** *p* < .001.

β = Standardized linear beta-weight, OR = Odds-ratio (Poisson regression), *R* ^*2*^ = adjusted *R*-square.

^1^ Fully adjusted model with the following covariates included (columns adj β/adj OR): age, BMI, BMI^2^, marital status, educational status, occupation, socio-economy, physical activity, alcohol use (frequency and amount).

### 3.3 Adjustment to violence and granting of disability pension (DP)

In the crude analyses, the rate of DP was comparable in the resilient and unexposed clusters, whereas the vulnerable clusters had a significantly higher risk of early DP (Table 5). Adjusting for the number of violent events and the covariates did not change these findings for women. However, the risk of granting early DP was now smaller for men in the resilient as compared to the unexposed cluster. A higher number of experienced psychological threats predicted a higher rate of DP among men but not among women, whereas physical violence had no contribution.

**Table 5:** Adaption Status and Disability Pensions.

	Women *n* = 6913			Men *n* = 6048		
	OR	OR	Adj OR (*CI* 95%)	OR	OR	Adj OR (*CI* 95%)
Adaptation status						
Resilient	.99	.91	**1.04** (.83–1.31)	.86	.67**	**.72** * (.53–.98)
Vulnerable	1.31**	1.17	**1.30** * (1.02–1.65)	1.41*	1.04	**1.07** (.74–1.55)
Highly vulnerable	3.20***	2.76***	**2.29** *** (1.48–3.57)	3.59***	2.49*	**2.29** * (1.02–5.12)
Unexposed			1 (ref)			1 (ref)
No. events (range)						
Threats (0–3)		1.06	1.17 (.94–1.45)		1.40*	1.49** (1.12–1.99)
Physical (0–3)		1.23	1.16 (.90–1.48)		1.19	1.21 (.88–1.66)
Covariates ^1^						

*Notes*.

* *p* < .05,

** *p* < .01

*** *p* < .001.

OR = Odds-ratio.

^1^ Fully adjusted model with the following covariates included (column adj OR): age, BMI, BMI^2^, marital status, educational status, socio-economy, physical activity, alcohol use (frequency and amount).

## Discussion

The present population-based survey examined relationships between exposure to violence and mental health (depressed/anxious mood), symptoms of severe musculoskeletal pain and early granting of disability pension. The study examined two types of violent events: psychological violence (threats/feeling tormented) and actual physical violence (beaten/kicked).

Our first hypothesis that psychological violence represents a stronger health hazard than actual violence was supported. Those reporting more psychological violence reported poorer mental health than those reporting physical violence. The same findings emerged for musculoskeletal pain, as well as for early disability pension among men. The negative role of physical violence was minor in comparison. Interpersonal, intentional violence that usually includes threats thus represents a health hazard as reported previously [2, 3]; however, the present study indicated that it affected all health and functional outcome variables.

The second hypothesis received support, as subjects classified as resilient showed a comparable level of job functioning and good mental health as unexposed subjects. Men classified as resilient even showed a slightly reduced risk of early disability pension compared to unexposed men. The resilience group, however, showed slightly elevated symptoms of severe musculoskeletal pain, as the hypothesis of *allostasis* would predict [33–35].

The third hypothesis that vulnerable subjects would show the poorest health across all outcome variables was also supported.

The present study also indicates a dose-response relationship between violence and health, particularly for threats of violence. Subjects experiencing a higher number of such events had an increased risk of musculoskeletal pain and particularly poorer mental health, which is in line with most other studies [12, 13, 18, 45]. Moreover, the coefficients for exposure to violence in childhood were generally higher than during other life epochs, thus restating the importance of preventing childhood abuse or violence [46–50].

The Adverse Childhood Experiences (ACE) study involving more than 17,000 subjects found a consistent relationship between ACE and mental health problems (e.g., drug abuse, depression and suicide) as well as physical diseases (e.g., ischemic heart, liver and sexually transmitted diseases) [51]. The number of ACEs also seemed to form a dose-response relationship with, for example, lifetime risk of depression [50] akin to what was reported in the present study. The finding of more musculoskeletal pain following exposure to childhood violence also agrees with previous reports [52].

### 4.1 Resilience in the face of violence

The majority of subjects experiencing violence do not develop lasting mental health problems [27]. In the present study, six out of 10 subjects reported no noticeable negative health impact following violent encounters, which corresponds to findings by others [53, 54]. Exposed but resilient subjects functioned equally well as unexposed subjects with regard to mental health, and even slightly better with regard to job functioning (lower risk of disability pensioning). Experience with hardships in life, such as violence, may have the paradoxical effect of providing future protection against later mental health problems given that coping is possible [55]. As the resilient subjects described themselves as “not affected,” such a functional coping response might underlie their good mental health. However, the lack of assessed coping variables in the study makes this assumption unsubstantiated. A negative finding was that resilient subjects showed slightly higher odds of musculoskeletal pain compared to unexposed subjects. These findings may indicate that resilient subjects develop more health complaints that are possibly caused by the increased *allostatic* load [35] following encounters with violence. This makes sense, as psychological violence, which represents a more chronic stressor and thus a particular health hazard, may also generate stronger and longer-lasting neuroendocrine stress or immunological responses [32–34]. However, it seems that resilient subjects do not allow these physical ailments to overshadow their mental well-being or ability to function adequately by keeping their job.

### 4.2 Gender differences

Some gender differences were noted, as indicated by earlier reports [23, 25]. Men were 1.3 times more often exposed to violence in childhood compared to women (12.9% vs. 9.4%, respectively), whereas the ratio was 1.8 and in the opposite direction in adulthood (4.9% vs. 9.0%, respectively). These age-related differences coincide with a large Swedish national public health report from 2012 [56]. Women thus suffer more health hazards than men do in adult years. Moreover, the mental health of women was more negatively impacted by exposure to violence than men’s mental health, but this difference was minor (*R* ^*2*^ = 7.5% vs 6.7%, respectively). The current study nevertheless showed a trend for a slightly raised vulnerability among women, which a population study in a neighboring country (Sweden) also indicated [23]. Exposure to psychological violence in childhood was more reliably related to poorer health among women than men, particularly with regard to musculoskeletal pain. Women also dominated the vulnerable adaptation clusters, which indicate that the depressogenic effects of violent encounters are in general higher for women than for men.

An opposite gender difference in our study was that men’s health (both mental and physical pain) was slightly more negatively associated with physical violence in childhood compared to women. As the present study did not specify the kind of violence experienced, the reason for the difference is unknown. Men are more often violated non-domestically [57] whereas women more often experience interpersonal violence at home [23]. However, since it the perpetration happened during the childhood years, it may have been of an intentional and interpersonal character, which may be worse [3] for men.

### 4.3 Implications for prevention

The negative relation between childhood violence and poor adult health is well established, yet, rarely acknowledged in general medical practice or emergency departments. Physicians infrequently ask for history of abuse and only 2–5% of patients tell the physician without being asked [58]. In a Norwegian study, physical abuse was only identified among 0.4% of the referred adolescent patients [59]. The failure of health personnel to ask direct question about abuse or violence may even contribute to the patients’ tendency to avoid bringing up these disturbing experiences, thus indirectly contributing to sustained health impairments.

To prevent health problems as a result of violence, barriers to overcome [60] include a proper institutional policy for effective screening/identification and a policy allocating sufficient time for high-quality consultations. Health personnel need privacy and time to conduct complete patient histories, adeaute training in clinical communication skills to facilitate detection of signs and symptoms of violence [1].

### 4.4 Strengths and limitations of the study

An obvious strength of the present study is the large sample size, the epidemiological design and the high quality control of the data collection process. The attendance rate (66%) was good compared with other large health surveys in Norway, such as the HUNT (Health Study of Nord-Trøndelag) study (56% response rate, *N* = 59,000). In a large epidemiological study in Norway, the non-response bias was small for correlation coefficients compared to prevalence point estimates [61]. As the main research questions of the current study concerned correlations, the impact of non-response should be minimal.

On the other hand, the large sample size only allowed self-report as the basis for classifying subjects as unexposed (i.e., normal or healthy), resilient, or vulnerable. Here, large-scaled epidemiological studies have a disadvantage compared to clinical studies, where it is feasible to conduct structured diagnostic interview methods to increase the validity [26, 54] of clustering subjects into resilient and vulnerable groups. However, as the point prevalence of psychopathology in the population is low [62], the number of wrongly classified subjects should constitute only a very small group. As the precision of most reported coefficients was high (*p*’s < .001), particularly with regard to mental health, a correction of any bias incurred by the self-report method is not expected to nullify the significance of the present findings. Moreover, as the resilience group showed an even smaller risk of early disability pension compared to the unexposed subjects, a considerable number of misclassifications would be required to change this direction qualitatively.

The use of paper-and-pencil methods to retrospectively collect information about exposure to violence has some well-known limitations as well as benefits. The main concern relates to under- versus over-reporting of violence. The poorer statistical power that goes along with underreporting was not an issue here. In addition, as the questionnaire method does not trigger embarrassment or fear of social stigma as much as face-to-face methods [63], the questionnaire approach should identify true cases acceptably well [64]. Rather, over-reporting may be a larger problem due to the global format of the two violence questions. The respondents’ personal interpretation of what constitutes “violence” may encompass a wider range of milder cases [65]. To what extent this was the case in the present study is unknown.

## Conclusion

The present study confirmed that exposure to violence increases the risk of poorer mental health in terms of more depression/anxiety, but also somatic health in terms of increased symptoms of severe musculoskeletal pain. The effect sizes of the relationships were in the small range, as oft reported. Psychological violence was more negatively related to poorer health than actual physical violence. In addition, a dose-response relationship was observed; a higher number of exposure to violent events implied increasingly poorer health, particularly mental health. About six of 10 subjects did not report a noticeable negative impact following violence in terms of health outcomes, hence indicating good adjustment or *resilience*. The resilient group had equally good mental health as subjects not reporting any violence but had slightly elevated symptoms of severe musculoskeletal pain. On the other hand, their risk of early disability pension was lower. Resilience to violence is quite prevalent, for which future studies should examine possible reasons.
