# Correction: Violence Affects Physical and Mental Health Differently: The General Population Based Tromsø Study

**DOI:** 10.1371/journal.pone.0210822

**Published:** 2019-01-10

**Authors:** Oddgeir Friborg, Nina Emaus, Jan H. Rosenvinge, Unni Bilden, Jan Abel Olsen, Gunn Pettersen

The data set underlying the findings described in this article was uploaded to the Dryad Digital Repository in error and has been removed. As a result, the Data Availability statement is incorrect. The correct statement is: The data underlying this study is third party and cannot be made publicly available. Any researcher may apply to the management of the Tromsø research and health study and obtain the same raw dataset as we did. The creation of sum- or mean scores for the relevant questionnaire variables/subscales would be at the discretion of the applicant. We, as authors, did not have any special access privileges. Data requests can be made at https://en.uit.no/om/enhet/artikkel?p_document_id=80172&p_dimension_id=88111
